# Next-generation bacteriophage therapeutic systems: CRISPR-based engineering, near-infrared bioimaging, and precision strategies for treating multidrug-resistant and extensively drug-resistant bacterial infections

**DOI:** 10.3389/fmicb.2026.1748742

**Published:** 2026-04-01

**Authors:** Zahra Zahid Piracha, Umar Saeed

**Affiliations:** 1 Széchenyi István University, Győr, Hungary; 2 University College, Korea University, Seoul, Republic of Korea; 3 Institute of Graduate Studies and Research, Cyprus International University, Nicosia, Cyprus

**Keywords:** bacteriophage therapy, biofilm disruption, CRISPR-engineered bacteriophages, extensively drug-resistant bacteria, jumbo phages, multidrug-resistant bacteria, near-infrared bioimaging, phage pharmacokinetics

## Abstract

The rapid rise of multidrug-resistant and extensively drug-resistant bacterial infections has renewed interest in bacteriophages as adaptable, targeted antimicrobials. Recent advances in phage engineering, including CRISPR-based approaches, now make it possible to refine host range, strengthen lytic performance, and deliver genetic payloads that target clinically important resistance determinants such as *blaNDM*, *mecA*, and *mcr-1*. In parallel, jumbo phages with large genomes often encode additional functions that support replication and biofilm disruption, offering practical advantages in densely structured infections where antibiotics perform poorly. A second limitation in phage translation has been measurement: in most settings, dosing and treatment duration remain guided by indirect endpoints rather than real-time information on distribution and activity. Near-infrared bioimaging addresses this gap by enabling noninvasive tracking of infection burden and phage kinetics *in vivo* through bacteriophytochrome-derived reporters, including iRFPs, miRFPs, and PAiRFPs. In this review, we bring these developments together and discuss how CRISPR-enabled phage engineering, jumbo-phage biology, and near-infrared readouts can be integrated into a precision framework that is measurable, adaptable, and clinically interpretable. We examine evidence across major drug-resistant pathogens, including *Pseudomonas aeruginosa*, *Acinetobacter baumannii*, *Klebsiella pneumoniae*, methicillin-resistant *Staphylococcus aureus*, vancomycin-resistant enterococci, *Burkholderia cepacia*, and *Mycobacterium abscessus*. We also summarize practical constraints that remain central to clinical translation, manufacturing quality, host immune neutralization, and regulatory variability, and outline a realistic development pathway in which engineered phages and companion diagnostics progress from animal models to carefully defined clinical indications. Together, these advances support a shift from empirical phage use toward a more standardized, data-driven approach to treating drug-resistant infections.

## 1 Introduction

Escalating antimicrobial resistance among World Health Organization priority pathogens has renewed global interest in bacteriophages, shifting their perception from historical biological curiosities to adaptable and highly targeted antimicrobial agents (WHO, 2017; CDC, 2019; O’Neill, 2016; Laxminarayan et al., 2016). The long-standing reliance on antibiotics as the cornerstone of modern medicine is increasingly undermined as multidrug-resistant and extensively drug-resistant bacteria continue to spread across both hospital and community settings. Life-threatening infections caused by *Pseudomonas aeruginosa*, *Acinetobacter baumannii*, *Klebsiella pneumoniae*, drug-resistant Enterobacterales, *Staphylococcus aureus*, and *Enterococcus faecium* together account for more than one million deaths annually, placing routine surgical procedures, cancer chemotherapy, organ transplantation, and intensive care practice under growing threat (WHO, 2017; CDC, 2019). These organisms deploy layered resistance strategies, including *β*-lactamases, carbapenemases, aminoglycoside-modifying enzymes, and high-capacity efflux systems. Their ability to establish dense, structured biofilms further reduces antibiotic penetration and effectiveness, rendering many antimicrobial classes functionally inadequate (O’Neill, 2016; Laxminarayan et al., 2016). The global rise of carbapenem-resistant Enterobacterales and colistin-resistant *A. baumannii* has therefore become a defining signal of the emerging post-antibiotic era.

Within this context, bacteriophages have re-emerged as biologically responsive therapeutics with properties distinct from conventional antimicrobials. Unlike static antibiotics, phages amplify selectively at the site of infection, using bacterial density as an intrinsic cue that concentrates activity where it is most needed (Gootenberg et al., 2018). Following adsorption, phages redirect host cellular machinery toward viral replication, culminating in bacterial lysis and the release of progeny that extend the therapeutic effect. Many clinically relevant phages encode depolymerases and endolysins capable of degrading extracellular polymeric substances, thereby weakening biofilm architecture and improving access to otherwise shielded bacterial populations. Beyond direct killing, phage pressure can shape bacterial evolutionary trajectories. Resistance-associated receptor mutations may reduce virulence or compromise efflux activity, as illustrated by phage OMKO1, which drives predictable re-sensitization of resistant strains to antibiotics (Abedon et al., 2011; Sulakvelidze et al., 2001; Yosef et al., 2015; Chan et al., 2016). When applied with appropriate timing, this evolutionary trade-off underpins phage–antibiotic synergy, resulting in more effective bacterial clearance than either approach alone (Chen et al., 2018; Schooley et al., 2017; Turner et al., 2021).

The renewed clinical relevance of phage therapy is closely linked to advances in molecular biology, synthetic genomics, and systems-level design. CRISPR–Cas systems, initially characterized as bacterial adaptive immune mechanisms, have been repurposed as precise genomic tools for engineering phages with enhanced therapeutic function. Phages armed with CRISPR payloads can selectively target resistance determinants such as *blaNDM*, *mecA*, and *mcr-1*, enabling strain-specific killing or restoration of antibiotic susceptibility within complex microbial communities (Chaikeeratisak et al., 2017; Filonov et al., 2011; Shcherbakova and Verkhusha, 2013). This capability represents a fundamental shift in antimicrobial strategy, positioning phages not only as lytic agents but also as vehicles for targeted genetic intervention.

Parallel progress in optical biotechnology has further expanded the phage therapeutic toolkit. Bacteriophytochrome-derived near-infrared fluorescent proteins, including iRFPs, miRFPs, and PAiRFPs, permit deep-tissue, noninvasive imaging with minimal background autofluorescence (Piatkevich et al., 2013; Shcherbakova et al., 2015; Ntziachristos, 2010; Köhler et al., 2023). These reporters penetrate biological barriers more effectively than visible-range fluorophores, enabling *in vivo* visualization of infection burden, phage distribution, and clearance dynamics. In animal models, near-infrared imaging has allowed real-time assessment of phage pharmacokinetics and pharmacodynamics, supporting more informed decisions regarding dose, timing, and route of administration. Together, these imaging approaches offer a path toward integrating measurement and therapy, addressing one of the longstanding limitations of translational phage research.

This review brings together several rapidly advancing strands of phage research into a unified framework for precision phage therapeutics. It integrates pathogen-specific evidence spanning *Pseudomonas aeruginosa*, *Acinetobacter baumannii*, *Klebsiella pneumoniae*, drug-resistant Enterobacterales, methicillin-resistant *Staphylococcus aureus*, vancomycin-resistant enterococci, *Stenotrophomonas maltophilia*, *Burkholderia cepacia*, and *Mycobacterium abscessus*. Alongside these clinical contexts, the review highlights progress in jumbo phage discovery, rational phage cocktail design, CRISPR-enabled phage engineering, and the development of delivery platforms such as inhalable formulations, hydrogels, and liposomal carriers. Emerging regulatory approaches are also considered, including Belgium’s magistral model, which permits personalized phage preparations under pharmacy-led, quality-controlled oversight. In parallel, the interface between phage therapy and modern molecular diagnostics is examined, with particular attention to CRISPR-based detection systems such as DETECTR and SHERLOCK.

The motivation for this synthesis arises from a clear and increasingly pervasive clinical reality: multidrug-resistant and extensively drug-resistant infections are no longer confined to high-acuity tertiary centers but have become routine challenges across diverse healthcare settings worldwide (Lister et al., 2009; Poole, 2011; Seed, 2015). *Pseudomonas aeruginosa* illustrates the complexity of this problem, combining *β*-lactamase production, loss of the OprD porin, mutator phenotypes, and dominance of the MexAB–OprM efflux system to withstand even recently introduced agents such as ceftazidime–avibactam and cefiderocol (Hyman, 2019; Chan et al., 2018; Burmeister et al., 2020). In this setting, bacteriophages offer a fundamentally different therapeutic logic. Their activity is highly specific, minimizes disruption of the commensal microbiota, and is intrinsically self-limiting once the bacterial host is eliminated. In addition, phage-encoded enzymes can disrupt biofilm architecture, and phage-driven evolutionary pressure can reshape resistance pathways in ways that restore antibiotic susceptibility (Labrie et al., 2010; Westra et al., 2014; Comeau et al., 2007). When deployed in a rational, complementary manner, phage therapy therefore extends beyond its historical role as a salvage option and instead functions as a systems-level partner capable of enhancing antimicrobial performance and altering resistance trajectories within precision medicine frameworks.

## 2 Molecular and evolutionary mechanisms underpinning precision phage therapy

### 2.1 Lytic programming, molecular targeting, and enzymatic remodeling of biofilms

The clinical utility of bacteriophages is shaped by three linked properties: replication strategy, receptor specificity, and the ability to function within biofilm-structured infections. Together, these factors determine killing efficiency, resistance emergence, and predictable evolutionary trade-offs. Strictly lytic phages within the class *Caudoviricetes* remain preferred for therapeutic use because their infection cycle is direct and ends in host lysis. By contrast, temperate phages require careful engineering or exclusion, including removal of integrases, excisionases, repressors, and toxin-associated elements, to reduce the risk of lysogeny and horizontal gene transfer (Abedon et al., 2011; Sulakvelidze et al., 2001; Turner et al., 2021; Hatfull, 2015).

Phages attach to bacterial hosts through defined surface structures, including lipopolysaccharide core components, capsular polysaccharides, type IV pili, flagella, and porins such as OprF and OprD. Many of these receptors contribute to virulence, nutrient uptake, or antibiotic resistance. As a result, receptor mutations that confer phage escape may impose fitness costs or alter susceptibility to antibiotics. This evolutionary coupling, often referred to as resistance steering, can be exploited when designing cocktails and combination regimens (Chan et al., 2018; Burmeister et al., 2020; Labrie et al., 2010; Westra et al., 2014; Hauser, 2009; Fortaleza et al., 2025).

A further advantage of many therapeutic phages is their capacity to disrupt biofilms. Tailspike depolymerases, capsular polysaccharide lyases, and endolysins can degrade extracellular polymeric substances and weaken biofilm architecture, improving access to bacteria embedded in deeper layers (Latka et al., 2017; Knezevic et al., 2013; Pires et al., 2016; Lin et al., 2017). These enzymes are also increasingly developed as standalone or hybrid antimicrobials, including engineered lysin-based agents and related enzybiotics, with the aim of improving penetration, supporting antibiotic diffusion, and reducing biofilm tolerance.

### 2.2 Jumbo phages and the phage nucleus: a compartmentalized strategy against bacterial immunity

Jumbo phages, typically defined by genomes exceeding 200 kilobases, encode expanded functional repertoires that support complex replication programs and greater control over host processes (Chaikeeratisak et al., 2017; Mendoza et al., 2020; Al-Shayeb et al., 2020; Hendrix, 2009; Malone et al., 2020; Chaikeeratisak et al., 2019). A distinctive feature described in several large phages infecting *Pseudomonas* is the formation of a nucleus-like protein compartment that encloses replicating phage DNA. By separating replication from the cytoplasm, this structure reduces exposure to host defenses, including CRISPR–Cas systems, restriction–modification pathways, and other antiviral enzymes.

Functionally, jumbo phages have shown strong lytic activity against highly drug-resistant pathogens, particularly *Pseudomonas aeruginosa* and *Acinetobacter baumannii*, and may retain activity in biofilm-rich environments where smaller phages and antibiotics are less effective (Al-Shayeb et al., 2020; Hendrix, 2009; Malone et al., 2020). Their development for therapeutic use, however, requires rigorous preclinical characterization. Genome sequencing and annotation are essential to exclude resistance genes, virulence factors, and lysogeny-associated modules. In addition, their larger capsids and genomes necessitate careful attention to manufacturing parameters such as packaging stability, batch-to-batch consistency, and endotoxin control (Turner et al., 2021; Chaikeeratisak et al., 2017; Filonov et al., 2011; Al-Shayeb et al., 2020). When these criteria are met, jumbo phages can serve as valuable chassis for precision phage therapy and may broaden the design space for durable anti-biofilm interventions.

### 2.3 Endolysins and artilysins: precision cell-wall disruptors

Endolysins are phage-encoded peptidoglycan hydrolases that play a central role in bacterial lysis during the terminal stage of the phage replication cycle. By degrading the cell wall from within, these enzymes trigger rapid osmotic rupture of the bacterial cell. When applied exogenously to Gram-positive bacteria, endolysins can bypass the requirement for phage infection altogether, as the absence of an outer membrane allows direct access to the peptidoglycan layer. In this context, they function as highly specific protein-based antibacterials with rapid bactericidal activity.

In Gram-negative pathogens, the outer membrane presents a major obstacle that limits the standalone use of native endolysins. This constraint has driven the development of artilysins—engineered constructs that combine catalytic endolysin domains with membrane-active elements such as cationic peptides, membrane-permeabilizing motifs, or cell-penetrating domains. These additions enable traversal of the outer membrane and extend lytic activity to otherwise refractory organisms, including *Pseudomonas aeruginosa*, *Klebsiella pneumoniae*, and *Acinetobacter baumannii* (Latka et al., 2017; Pastagia et al., 2011; Schuch et al., 2014; Briers et al., 2014; Defraine et al., 2016). In several experimental settings, artilysins have demonstrated additive or synergistic effects when combined with antibiotics such as colistin or carbapenems, partly by increasing membrane permeability and allowing dose reduction of companion agents—an important consideration for minimizing toxicity and resistance selection.

Exebacase (CF-301) remains the most advanced endolysin-derived therapeutic evaluated in clinical studies. Early-phase trials in *Staphylococcus aureus* bacteremia showed encouraging signals of efficacy, particularly when used alongside standard-of-care antibiotics. However, the subsequent Phase 3 trial did not meet its primary endpoint, a result that has been attributed in part to heterogeneity in infection source and insufficient consideration of biofilm-associated disease. These findings highlight a broader challenge for protein-based bacteriolytics: clinical trial endpoints optimized for planktonic bloodstream infections may not adequately capture benefit in biofilm-dominated or device-associated settings (Fowler et al., 2024; Schuch et al., 2014; McCarthy, 2024). Future studies will likely require more tailored endpoint selection and stratification strategies that better reflect real-world infection biology.

### 2.4 CRISPR-enabled phage constructs and genetic re-sensitization

The adaptation of CRISPR–Cas systems for antimicrobial use has introduced new possibilities for selectively targeting bacterial pathogens at the genetic level. Phages, conjugative elements, and nanoparticle-based carriers have been engineered to deliver CRISPR-associated nucleases, including Cas9, Cas3, and Cas12a, directly into resistant bacteria. These systems enable sequence-specific cleavage of resistance determinants such as *blaNDM*, *mcr-1*, *mecA*, and *vanA*, resulting in targeted bacterial killing or loss of resistance phenotypes (Yosef et al., 2015; Chen et al., 2018; Bikard et al., 2014; Citorik et al., 2014; Pursey et al., 2018; Rodrigues et al., 2019; Yan and Bassler, 2019; Kiga et al., 2020; Ramachandran and Santiago, 2023). Unlike broad-spectrum antibiotics, CRISPR-enabled constructs can achieve strain-level precision while largely preserving surrounding commensal microbiota.

Beyond direct lethality, an important feature of these approaches is their capacity to remove plasmid-borne resistance genes or disrupt chromosomal resistance islands, effectively restoring antibiotic susceptibility within bacterial populations. For example, CRISPR systems targeting *mcr-1* have successfully reversed colistin resistance in *Escherichia coli* and *Klebsiella pneumoniae* in preclinical models, re-enabling the activity of a last-resort antimicrobial (Citorik et al., 2014; Pursey et al., 2018; Rodrigues et al., 2019). These findings support the concept of genetic re-sensitization as a complementary strategy to conventional killing, particularly in settings where resistance determinants are well defined.

Despite their promise, CRISPR-enabled phage constructs face several technical and translational challenges. Off-target cleavage, neutralization by endogenous anti-CRISPR proteins, immune recognition of CRISPR components, and variability in phage–host receptor interactions all require careful optimization at the design stage (Bondy-Denomy et al., 2013; Pawluk et al., 2018). In addition, the convergence of gene-editing technologies with replicating biological vectors raises regulatory and biosafety considerations that extend beyond those of conventional antimicrobials. As a result, regulatory frameworks increasingly emphasize traceability, genomic stability, and robust preclinical safety evaluation prior to clinical application.

Taken together, CRISPR-enabled phage engineering represents a conceptual advance in antimicrobial strategy. Its greatest potential may lie not in replacing existing therapies, but in complementing them, by selectively dismantling resistance mechanisms and restoring the effectiveness of antibiotics that have otherwise been rendered obsolete.

## 3 Translational therapeutic design: from bench logic to bedside precision

### 3.1 Cocktail architecture: orthogonality, coverage, and evolutionary foresight

The design of effective phage cocktails depends on more than simply expanding host coverage. Productive combinations require deliberate selection based on lytic performance, evolutionary robustness, and functional compatibility between constituent phages. Initial screening typically involves testing large panels of candidate phages, followed by prioritization according to efficiency of plating, activity across genetically diverse clinical isolates, and the ability to reduce bacterial burden within biofilms. A central design principle is receptor orthogonality. When individual phages target distinct bacterial structures—such as lipopolysaccharide core components, type IV pili, capsular polysaccharides, or outer membrane porins including OprF and OprD—bacteria are forced to negotiate multiple evolutionary constraints rather than a single escape route. This diversification substantially lowers the probability of simultaneous resistance and increases the fitness cost associated with phage escape (Hyman, 2019; Labrie et al., 2010; Westra et al., 2014; Mirzaei and Nilsson, 2015; Jault et al., 2019; Pirnay et al., 2018; Hesse et al., 2020).

Incorporating at least one phage that encodes a capsule-degrading depolymerase further strengthens cocktail performance by weakening biofilm barriers and improving access for both phages and companion antibiotics. However, phage–phage interactions must also be considered. Some combinations exhibit cooperative effects that enhance replication and killing efficiency, whereas others compete intracellularly and reduce overall activity. For this reason, empirical testing of phage compatibility remains an essential step prior to clinical application.

Equally important is the anticipation of evolutionary trade-offs. Mutations that allow bacteria to evade one phage often impair virulence traits or reduce the efficiency of resistance mechanisms such as efflux pumps. In many cases, these changes restore susceptibility to antibiotics that were previously ineffective. When anticipated and exploited deliberately, such predictable evolutionary costs can be used to construct cocktails that not only eliminate pathogens but also destabilize their resistance networks (Chan et al., 2018; Burmeister et al., 2020; Labrie et al., 2010; Westra et al., 2014; Lin et al., 2017).

### 3.2 Biofilm-first strategies for chronic and device-associated infections

Biofilm-associated infections represent a persistent clinical challenge because their dense extracellular matrix limits antimicrobial penetration and supports metabolically heterogeneous bacterial populations. In preclinical models of catheter-related infections, airway colonization, burn wounds, and other chronic settings, phage cocktails enriched with depolymerase-producing phages have consistently achieved meaningful reductions in bacterial burden.

These outcomes reflect the complementary actions of phages and antibiotics within structured communities. Depolymerases disrupt the extracellular polymeric substances that stabilize the biofilm, increasing permeability and exposing embedded bacteria. Phages can then replicate within the biofilm architecture itself, reaching bacterial subpopulations that are otherwise inaccessible to antibiotics. Once the matrix is compromised and bacterial physiology is perturbed by phage infection, antibiotics gain improved access and exert enhanced bactericidal effects (Chaudhry et al., 2017; Latka et al., 2017; Knezevic et al., 2013; Pires et al., 2016; Lin et al., 2017; González et al., 2018; Dickey and Perrot, 2019).

Timing remains a critical determinant of success. Therapeutic schedules must be aligned with the transient periods during which biofilm integrity is reduced and bacterial cells are most vulnerable. Achieving this requires careful adjustment of dose and sequence in both experimental systems and clinical protocols, rather than reliance on fixed or simultaneous administration.

### 3.3 Phage antibiotic combinations: the role of therapeutic timing

Phage–antibiotic combinations are increasingly recognized as a rational strategy for managing resistant infections, but their effectiveness depends strongly on how and when each component is delivered. The interaction is not simply additive. Certain antibiotics, including beta-lactams and fluoroquinolones, induce physiological changes such as cell elongation, membrane stress, or altered surface architecture that increase phage adsorption and replication efficiency (Comeau et al., 2007; Torres-Barceló et al., 2014; Chaudhry et al., 2017; Oechslin, 2018; Levin and Bull, 1996). Conversely, phage infection may reduce capsule thickness or downregulate efflux activity, thereby enhancing antibiotic susceptibility in surviving bacterial populations.

Accumulating experimental evidence supports a sequential rather than simultaneous treatment paradigm. In this approach, phages are administered first to reduce bacterial density and disrupt protective structures such as biofilms. Antibiotics are introduced subsequently, during a window when residual bacteria are stressed, metabolically altered, and less capable of mounting coordinated resistance responses. Staggered dosing, often involving smaller and repeated administrations, has been shown to limit antagonistic interactions, reduce immune activation, and slow the emergence of resistance compared with single-dose or fully concurrent regimens (Comeau et al., 2007; Torres-Barceló et al., 2014; Chaudhry et al., 2017; Oechslin, 2018; Levin and Bull, 1996). As a result, timing has emerged as a key therapeutic variable in combination therapy. It is increasingly incorporated into pharmacokinetic and pharmacodynamic modeling frameworks that aim to translate phage–antibiotic synergy from controlled experiments to clinically meaningful treatment strategies.

### 3.4 Routes of administration and the pharmacokinetic and pharmacodynamic complexities of living therapies

Route of administration is a major determinant of phage therapy performance because phages function as replicating biologics rather than fixed-dose chemicals. The delivered inoculum is only one component of exposure; tissue distribution, bacterial burden at the infection site, and host immune clearance jointly shape whether *in situ* amplification occurs and how long active phages persist.

For bloodstream infection and other systemic disease, intravenous delivery is generally the most direct approach. This route, however, places stringent demands on product quality and infusion practice. Endotoxin control is essential, and dosing strategies must consider both infusion kinetics and the possibility of rapid immune-mediated clearance. Clinical protocols therefore emphasize careful monitoring of safety markers alongside phage pharmacokinetic behavior, including amplification signals and clearance dynamics (Schooley et al., 2017; Aslam et al., 2020; Doub et al., 2020; Petrovic Fabijan et al., 2020).

For respiratory infections such as ventilator-associated pneumonia, cystic fibrosis, and bronchiectasis, nebulized delivery has the practical advantage of depositing phages directly into the airways. Therapeutic performance depends on aerosol characteristics, penetration through mucus and sputum, and the ability of phages to remain viable and replicate within airway secretions. Inhalation strategies also require attention to formulation stability and device compatibility, as these variables can substantially affect the effective dose reaching distal airways (Köhler et al., 2023; Van Nieuwenhuyse et al., 2022; Ferry et al., 2022).

Topical and locoregional delivery is particularly attractive for infections dominated by biofilm architecture, including chronic wounds, device-associated urinary tract infection, prosthetic joint infection, and osteomyelitis. These approaches allow high local phage concentrations with limited systemic exposure (Jault et al., 2019; Doub et al., 2020; Morris et al., 2019).

Phage pharmacokinetics differ fundamentally from conventional drugs. Multiplicity of infection should not be equated with the administered dose, because effective exposure depends on bacterial density, structural accessibility of the infection niche, local diffusion constraints, and immune activity. Neutralizing antibodies can emerge during prolonged or repeated therapy and may reduce efficacy, particularly for systemic regimens. Several mitigation strategies are under investigation, including serotype rotation, chemical modification such as polyethylene glycol conjugation, and formulation approaches such as liposomal encapsulation aimed at prolonging circulation time and reducing immune recognition (Oechslin, 2018; Rodrigues et al., 2019; Yan and Bassler, 2019; Aslam et al., 2020; Doub et al., 2020; Petrovic Fabijan et al., 2020; Hodyra-Stefaniak et al., 2015). Collectively, these considerations support the need for route-specific pharmacokinetic and pharmacodynamic frameworks that treat phages as living therapeutics rather than simple antimicrobials.

## 4 Pathogen-specific dossiers: strategic deployment of phage therapy in multidrug-resistant and extensively drug-resistant infections

### 4.1 Pseudomonas aeruginosa

*Pseudomonas aeruginosa* remains among the most treatment-refractory pathogens because resistance mechanisms frequently converge within the same clinical isolate. These include dominance of the MexAB–OprM efflux system, loss-of-function changes in OprD that reduce carbapenem uptake, an array of chromosomal and plasmid-mediated beta-lactamases, and alginate-rich biofilms that restrict antimicrobial penetration and blunt host clearance (Lister et al., 2009; Poole, 2011; Hauser, 2009). Such features are particularly relevant in cystic fibrosis, ventilator-associated pneumonia, and major burn injury, where bacterial burden is high and treatment must contend with both dense biofilm structure and ongoing inflammatory damage.

The clinical evidence base for phage therapy in *P. aeruginosa* is still shaped largely by compassionate-use reports and small series, but these experiences provide useful mechanistic and practical signals. Aerosolized personalized phage administration has been associated with detectable airway replication and clinical improvement in selected cases of chronic pulmonary infection, including Kartagener syndrome (Köhler et al., 2023). In systemic disease, prolonged intravenous phage therapy administered alongside beta-lactams has been reported to clear infection in highly resistant settings, and phage-resistant derivatives in some cases exhibit reduced virulence, consistent with evolutionary trade-offs that may be therapeutically exploitable (Van Nieuwenhuyse et al., 2022).

Dual-route strategies, combining inhaled and intravenous administration, have been used in critically ill burn patients with pulmonary and bloodstream involvement, sometimes alongside adjunct immunomodulation such as interferon-gamma (Ferry et al., 2022). The potential contribution of such adjuncts remains difficult to isolate from source control and antibiotic therapy, and these reports should be interpreted as hypothesis-generating rather than definitive evidence. Similarly, jumbo phages with large genomes have shown strong activity against difficult-to-treat *P. aeruginosa* strains in experimental systems and in selected clinical contexts; however, broader clinical validation is still needed, and manufacturing consistency and genomic safety screening remain central considerations (Al-Shayeb et al., 2020; Hendrix, 2009; Malone et al., 2020; Chaikeeratisak et al., 2019).

Phage OMKO1 illustrates a rational design concept relevant to *P. aeruginosa*: targeting the OprM component of an efflux-associated structure can drive resistance pathways that reduce efflux efficiency and restore antibiotic susceptibility (Chan et al., 2018; Burmeister et al., 2020). Building on this principle, an optimized therapeutic cocktail for *P. aeruginosa* would typically prioritize receptor diversity, such as phages targeting lipopolysaccharide structures, type IV pili, and outer membrane porins including OprD or OprF, together with at least one depolymerase-producing phage to support biofilm penetration. Antibiotic partners, including ciprofloxacin, meropenem, ceftolozane–tazobactam, or colistin, should be selected using *in vitro* synergy testing and interpreted through route-specific pharmacokinetic considerations. In general, inhalation remains the most direct strategy for airway-dominant disease, while intravenous administration is required when infection is systemic or bacteremic (Hyman, 2019; Chan et al., 2018; Burmeister et al., 2020; Labrie et al., 2010; Westra et al., 2014; Comeau et al., 2007; Torres-Barceló et al., 2014; Chaudhry et al., 2017; Oechslin, 2018; Latka et al., 2017; Van Nieuwenhuyse et al., 2022).

### 4.2 *Acinetobacter baumannii* (carbapenem-resistant *Acinetobacter baumannii*)

Carbapenem-resistant *Acinetobacter baumannii* has become a persistent challenge in intensive care units, where ventilator-associated pneumonia, bloodstream infection, and complicated intra-abdominal disease can progress rapidly and leave few reliable antibiotic options. Clinical interest in phage therapy intensified after a compassionate-use report in 2017 described successful treatment of a critically ill patient using a tailored phage approach administered by combined systemic and intraperitoneal routes (Schooley et al., 2017; Aslam et al., 2020; Doub et al., 2020; Petrovic Fabijan et al., 2020; LaVergne et al., 2018; Hatab et al., 2023). Since then, additional case series and early clinical experiences have supported the feasibility of phage therapy for carbapenem-resistant *A. baumannii*, particularly when integrated with source control and used alongside active companion antibiotics. Agents commonly used in these combination strategies include tigecycline and rifampin, and more recently sulbactam–durlobactam in settings where it is available and appropriate.

Route selection is driven by infection site. For ventilator-associated pneumonia, nebulized delivery is often favored to maximize airway deposition, while intravenous administration is used when infection is systemic or accompanied by bacteremia. Device-associated infection remains a difficult niche because intraluminal biofilms can sustain relapse even when systemic therapy appears adequate. In this context, phage lock therapy has emerged as a pragmatic adjunct strategy aimed at achieving high local titers within the device lumen while limiting systemic exposure (Doub et al., 2020; Hatab et al., 2023). As with other pathogens, careful genomic screening and quality control are essential to exclude undesirable genetic cargo and ensure batch consistency before clinical use.

### 4.3 *Klebsiella pneumoniae* (extended-spectrum beta-lactamase producers and carbapenem-resistant strains)

*Klebsiella pneumoniae* is distinguished by its polysaccharide capsule, a major virulence determinant that also creates a physical and functional barrier to immune clearance, antibiotic activity, and phage adsorption. This biology makes capsule-directed phage strategies particularly relevant. Depolymerase-producing phages can enzymatically degrade capsule polymers, improving access to the bacterial surface and enhancing phage penetration into structured communities. In experimental systems, these capsule-active phages have shown synergy with beta-lactam/beta-lactamase inhibitor combinations and other antibiotics, including ceftazidime–avibactam and meropenem–vaborbactam, with stronger effects typically reported when treatment is initiated early in infection models (Lin et al., 2014; Pan et al., 2013; Hoyles et al., 2022; Aleshkin et al., 2021; Slopek et al., 1987; D’Andrea et al., 2017).

From a translational perspective, therapeutic design is complicated by capsule diversity. Effective intervention often requires a multiphage cocktail that covers several capsule types and clinically important lineages, including high-risk clones such as ST258. This is also a pathogen group where strict genomic vetting is non-negotiable. Environmental *Klebsiella* phages can carry mobile genetic elements, and any candidate intended for therapeutic use must be screened to exclude antimicrobial resistance determinants, virulence-associated loci, and lysogeny-related modules (Turner et al., 2021; Hatfull, 2015). These considerations support a rational approach in which capsule coverage, depolymerase activity, and manufacturing-grade genomic safety form the core criteria for phage selection in *K. pneumoniae* infection.

### 4.4 Enterobacterales, including uropathogenic *Escherichia coli* and extended-spectrum beta-lactamase producers

Uropathogenic *Escherichia coli* and other extended-spectrum beta-lactamase–producing Enterobacterales remain leading causes of recurrent and complicated urinary tract infection, particularly when prior antibiotic exposure narrows options and biofilm-like communities persist within the bladder or along devices. Early clinical evaluation of intravesical phage delivery suggests that local administration can be feasible and generally well tolerated, while also offering practical advantages for urinary infections because high titers can be achieved at the site of disease with limited systemic exposure.

Alongside conventional lytic cocktails, gene-targeted approaches are being explored in which engineered phages deliver clustered regularly interspaced short palindromic repeats–associated nucleases to selectively eliminate strains carrying key resistance determinants, including *bla*NDM and *bla*CTX-M variants (Bikard et al., 2014; Citorik et al., 2014; Pursey et al., 2018; Rodrigues et al., 2019; Leitner et al., 2021; Kortright et al., 2019; Howard-Jones et al., 2022). Importantly, most of this work remains preclinical or early translational, and the language in this section should reflect that these platforms are still evolving rather than already positioned for routine clinical use. A parallel development with immediate practical relevance is rapid susceptibility matching. Microfluidic “phagogram” systems can support same-day selection of active phages against a patient isolate, an approach that aligns closely with the operational needs of complicated urinary tract infection management, where delayed effective therapy can drive recurrence and escalation.

### 4.5 *Staphylococcus aureus* resistant to methicillin

Methicillin-resistant *Staphylococcus aureus* continues to cause difficult-to-clear infections in which biofilms and tissue sequestration dominate the clinical course, including prosthetic joint infection, osteomyelitis, and persistent bacteremia. In this setting, phage-based strategies are attractive because they can be deployed locally, incorporated into multimodal regimens, and designed to prioritize biofilm disruption. Engineered phages delivering clustered regularly interspaced short palindromic repeats–associated payloads have shown selective killing of methicillin-resistant *S. aureus* in animal models by targeting resistance or survival determinants, but these data should be framed as experimental rather than established clinical practice.

Phage-derived lysins provide a more mature translational path in this organism because they can act independently of phage replication. Lysins such as LysK and exebacase have demonstrated strong activity against methicillin-resistant *S. aureus* and can weaken biofilms, with additive effects reported when combined with standard agents such as rifampin or daptomycin (Pastagia et al., 2011; Fowler et al., 2024; Schuch et al., 2014; McCarthy, 2024; Fenton et al., 2010; Chan et al., 2013; Tkhilaishvili et al., 2019). The divergence between encouraging earlier clinical signals and the later failure to meet a primary endpoint in a phase three setting reinforces a practical point for the field: endpoints optimized for bloodstream sterilization may not adequately capture benefit in biofilm-driven disease, and trial design must better reflect infection origin, device involvement, and biofilm burden.

### 4.6 Enterococci resistant to vancomycin

Enterococci resistant to vancomycin remain difficult to manage in endocarditis, intra-abdominal infection, and central-line, associated bacteremia, particularly when host factors or device dependence increase relapse risk. Lytic phages active against *Enterococcus faecium* and *Enterococcus faecalis* have been described across multiple morphological families, and compassionate-use experiences support the feasibility of incorporating phages into salvage regimens in selected cases. Clinical reports of successful clearance exist, but susceptibility testing remains essential because enterococcal phage host range can be narrow, and resistance may emerge under monotherapy pressure.

Where phages are used, they are typically positioned as an adjunct to standard antibacterial therapy rather than a substitute. Combination with agents such as linezolid or daptomycin is commonly emphasized to reduce relapse risk and to maintain bactericidal pressure while phage activity evolves *in vivo* (Jault et al., 2019; Alrafaie et al., 2024; Kropinski et al., 2012; Fish et al., 2016; Ujmajuridze et al., 2018). As with other organisms, the most defensible framing is that the evidence base is still limited and heterogeneous, and that broader conclusions will require standardized dosing, defined endpoints, and controlled clinical evaluation.

### 4.7 *Stenotrophomonas maltophilia* and the *Burkholderia cepacia* complex

*Stenotrophomonas maltophilia* and the *Burkholderia cepacia* complex are clinically important in cystic fibrosis and in immunocompromised hosts, where intrinsic resistance, biofilm formation, and airway persistence restrict the effectiveness of conventional antibiotics. The published experience with phage therapy in these pathogens remains smaller than for *Pseudomonas* or *Staphylococcus*, but the direction of evidence is consistent: receptor-diverse phage cocktails, often delivered by inhalation, are being explored as adjunct approaches in scenarios where antimicrobial options are limited.

Because these infections are frequently polymicrobial and occur in structurally abnormal airways, phage strategies are typically integrated with antibiotic backbones rather than used alone. For *Stenotrophomonas*, companion therapy often includes trimethoprim–sulfamethoxazole or minocycline, while *Burkholderia* regimens are usually individualized and guided by susceptibility patterns and patient tolerance (Doub et al., 2020; Waters et al., 2017; Law et al., 2019; Chang et al., 2018).

### 4.8 Mycobacterium abscessus

*Mycobacterium abscessus* is among the most therapeutically challenging bacterial pathogens, particularly in individuals with cystic fibrosis and in lung transplant recipients, where infection can be progressive, recurrent, and poorly responsive to available antibiotics. Both intrinsic resistance mechanisms and rapid acquisition of additional resistance limit the effectiveness of standard multidrug regimens, often leaving clinicians with few viable options.

In this context, phage therapy has attracted attention following several compassionate-use cases in which engineered phages were employed to treat otherwise refractory disease. These phages were modified to ensure strictly lytic behavior and, in some cases, to broaden host range through genetic adaptation. Clinical responses reported in these settings—including reductions in bacterial burden and stabilization of lung function—represent an important proof of principle for the use of engineered phages against mycobacterial disease (Dedrick et al., 2019; Dedrick et al., 2021; Hatfull, 2018). At the same time, these experiences should be interpreted cautiously, as they reflect highly individualized interventions supported by intensive microbiological characterization and close clinical monitoring. Broader application will require standardized approaches to phage selection, manufacturing, and outcome assessment.

### 4.9 *Clostridioides difficile* (an adjacent application)

Although *Clostridioides difficile* is not typically categorized as a multidrug-resistant or extensively drug-resistant organism, it remains a major cause of morbidity through toxin-mediated colitis and frequent recurrence following antibiotic treatment. The pathophysiology of *C. difficile* infection—marked by disruption of the intestinal microbiota—makes it a relevant adjacent target for precision antimicrobial strategies that minimize collateral damage.

Phage-derived lysins, including variants of PlyCD, have demonstrated potent activity against toxigenic *C. difficile* strains in preclinical models, including biofilm-associated forms. These agents act directly on the bacterial cell wall and, in experimental settings, preserve commensal microbial communities more effectively than broad-spectrum antibiotics (Schuch et al., 2014; Nale et al., 2016). While randomized clinical data are not yet available, these findings support continued investigation of lysin-based approaches as a means of reducing recurrence and restoring microbiome stability in *C. difficile*–associated disease.

## 5 Imaging, diagnostics, and control loops: the near-infrared layer

The near-infrared spectral window, spanning approximately 650 to 900 nanometers, offers several practical advantages for biological imaging. Photons in this range penetrate tissue more deeply than visible light, are subject to reduced scattering, and generate lower levels of background autofluorescence from host tissues. These properties enable noninvasive, longitudinal visualization of infection dynamics and therapeutic responses in living systems, making near-infrared imaging well suited for studying phage–host–pathogen interactions in real time (Gootenberg et al., 2018; Filonov et al., 2011; Shcherbakova and Verkhusha, 2013; Piatkevich et al., 2013; Shcherbakova et al., 2015; Ntziachristos, 2010; Shcherbakova et al., 2016; Piatkevich et al., 2017; Shcherbakova et al., 2018; Filonov et al., 2011).

Central to this capability is a class of fluorescent reporters derived from bacterial phytochrome photoreceptors. These proteins use biliverdin, a naturally occurring product of heme catabolism, as their chromophore. Because biliverdin is endogenously available in mammalian tissues, near-infrared reporters do not require external substrates or dyes. This feature allows them to be genetically encoded into bacteria, phages, or host cells and monitored over time with minimal additional formulation or procedural complexity.

### 5.1 Molecular toolbox for near-infrared imaging

#### 5.1.1 Infrared fluorescent proteins

Infrared fluorescent proteins, including iRFPs and more recent miniaturized variants such as miRFP670nano and miRFP720, are monomeric, genetically encoded reporters optimized for brightness, photostability, and tissue penetration. Their relatively small size facilitates incorporation into bacterial or phage genomes, enabling applications such as labeling infection foci, tracking phage replication *in vivo*, and constructing reporter phages that function as biosensors of infection burden or therapeutic activity (Shcherbakova and Verkhusha, 2013; Piatkevich et al., 2013; Shcherbakova et al., 2015; Ntziachristos, 2010; Shcherbakova et al., 2016; Piatkevich et al., 2017; Shcherbakova et al., 2018; Filonov et al., 2011). In experimental models, these tools have provided quantitative insight into spatial and temporal aspects of phage therapy that are difficult to capture using conventional microbiological endpoints alone.

#### 5.1.2 Photoactivatable near-infrared fluorescent proteins

Photoactivatable near-infrared fluorescent proteins remain optically silent until triggered by illumination at a defined activation wavelength. This feature provides temporal and spatial control over signal generation, allowing investigators to “mark” a population of labeled phages or bacteria at a chosen time point and then follow their fate longitudinally. In practical terms, photoactivatable reporters can support pulse–chase style experiments that separate early localization and dissemination from later phases such as clearance after treatment. For instance, activation at the start of therapy followed by tracking of signal decay can provide a visual readout of response kinetics and the trajectory of infection resolution in vivo (Piatkevich et al., 2013; Piatkevich et al., 2013).

#### 5.1.3 Split reporters and near-infrared resonance energy transfer biosensors

Split fluorescent protein systems and near-infrared Förster resonance energy transfer biosensors extend imaging beyond simple presence–absence tracking by linking signal output to defined molecular events. In split designs, fluorescence is reconstituted only when two components are brought together, which can be engineered to occur during processes such as receptor engagement, enzymatic activation, or cleavage-dependent assembly. Near-infrared resonance energy transfer sensors similarly enable activity-linked readouts by reporting conformational changes or proximity changes between donor and acceptor pairs. When incorporated into reporter phage platforms, these strategies can distinguish passive distribution from productive infection, supporting more biologically meaningful visualization of where and when phage activity is occurring in vivo rather than where phage particles merely reside (Piatkevich et al., 2013; Shcherbakova et al., 2018; Greenwald et al., 2018).

#### 5.1.4 Photoacoustic imaging overlays

Near-infrared reporters can also be adapted for photoacoustic imaging, an approach that combines optical excitation with acoustic detection to improve depth and spatial resolution. In this modality, pulsed near-infrared light is absorbed by the contrast agent and converted into thermoelastic expansion, generating ultrasonic waves that can be detected and reconstructed into images. Compared with fluorescence alone, photoacoustic imaging can offer improved performance in deeper tissues, including organs such as the lungs and liver. Within experimental infection models, this overlay may enable assessment of phage distribution and therapeutic impact in settings where optical signal attenuation would otherwise limit interpretability, such as pneumonia, intra-abdominal abscesses, or central nervous system infections (Miao and Pu, 2016; Knox et al., 2017; Kasatkina et al., 2022).

#### 5.1.5 Linking near-infrared readouts with clustered regularly interspaced short palindromic repeats diagnostics

Clustered regularly interspaced short palindromic repeats–based diagnostic platforms, including specific high-sensitivity enzymatic reporter systems such as SHERLOCK and DETECTR, enable culture-independent detection of pathogens and resistance determinants with high analytical sensitivity. When paired with near-infrared readouts, these assays can be adapted to reduce background signal in complex clinical specimens such as blood, sputum, and urine, supporting clearer detection under conditions where optical noise is otherwise limiting (Chan et al., 2016; Chen et al., 2018; Myhrvold et al., 2018; Kellner et al., 2019; Broughton et al., 2020; Ackerman et al., 2020).

The conceptual advantage becomes most apparent when diagnostics are used to guide and then monitor precision phage interventions. As an example, a clustered regularly interspaced short palindromic repeats diagnostic assay could identify a resistance determinant such as blaNDM directly from a clinical sample; an engineered lytic phage carrying a targeting module directed at the same locus could then be selected as the therapeutic, and response could be monitored using a compatible reporter system. Framed appropriately, this represents a closed-loop strategy in which detection, therapeutic selection, and treatment monitoring are aligned around a shared genetic target, while remaining clear that most implementations are currently demonstrated in experimental or preclinical settings rather than routine clinical practice.

#### 5.1.6 From preclinical imaging to dose selection in humans

In experimental infection models, the use of engineered bacteria or bacteriophages carrying near-infrared reporters has made it possible to quantify aspects of phage behavior that are difficult to capture with conventional sampling alone. These include estimates of how quickly bacterial burden declines after treatment, patterns of tissue distribution that reflect phage tropism and replication niches, and time-resolved response profiles that distinguish early lag phases from peak activity and eventual resolution (Shcherbakova et al., 2015; Ntziachristos, 2010; Shcherbakova et al., 2018; Miao and Pu, 2016). Such readouts provide a dynamic view of treatment response rather than a single end-point measurement.

When interpreted carefully, these data can be used to develop mechanistic models that describe how phages propagate, persist, and are cleared in different tissues. After validation in animal systems, these models can inform hypotheses about dosing strategies in humans, particularly when combined with complementary, culture-independent measures such as quantitative polymerase chain reaction, circulating inflammatory markers, or clustered regularly interspaced short palindromic repeats–based diagnostics that capture similar information without relying on imaging. In this context, near-infrared imaging functions less as a visualization tool and more as a means of refining pharmacokinetic and pharmacodynamic reasoning, helping to frame questions about dose escalation, route selection, and combination scheduling in a rational, patient-specific manner rather than implying routine clinical deployment.

#### 5.1.7 Manufacturing, quality control, and regulatory considerations

**Figure 1:**
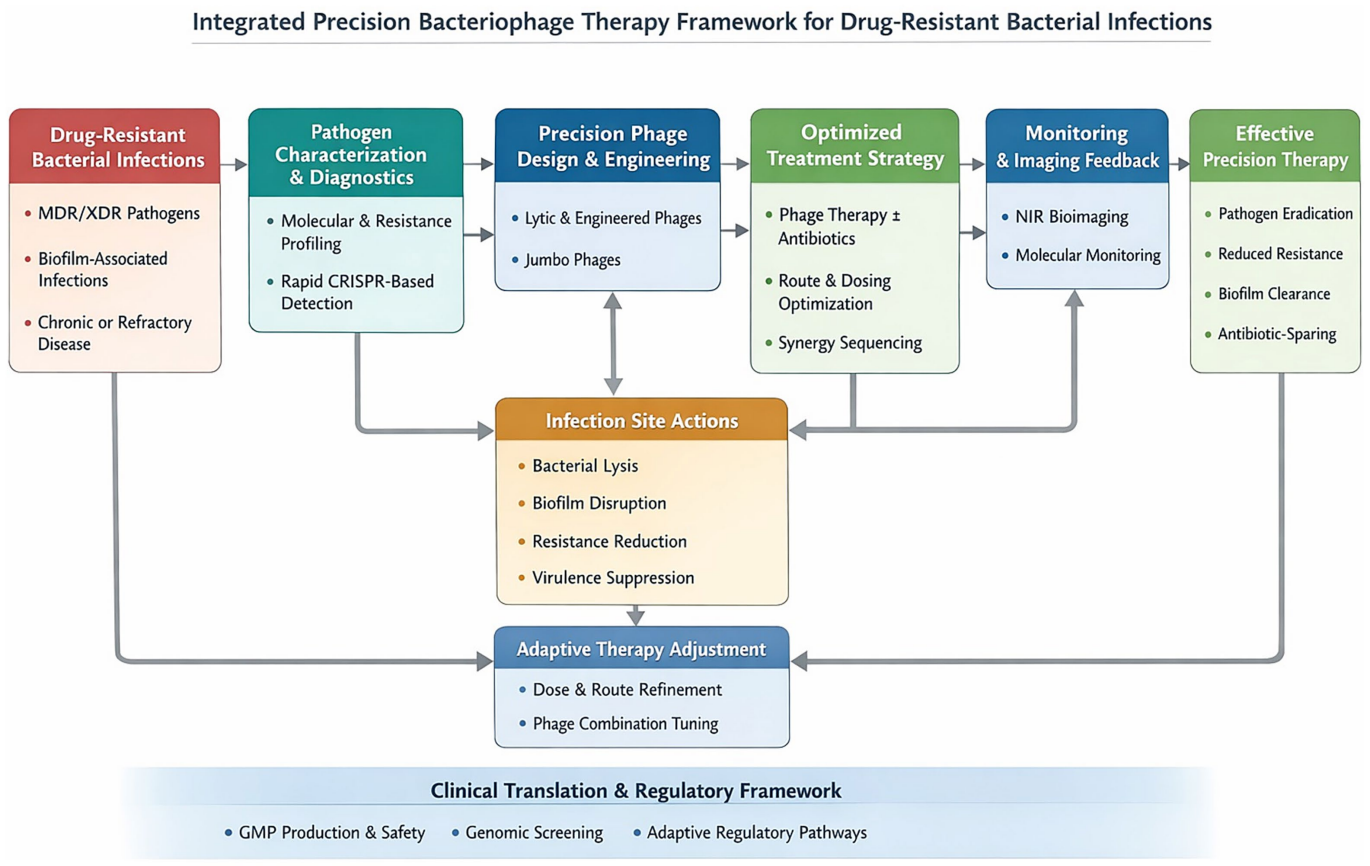
Integrated precision bacteriophage therapy framework for drug-resistant bacterial infections. This schematic illustrates a closed-loop precision bacteriophage therapy model designed to manage multidrug-resistant and extensively drug-resistant bacterial infections. The framework begins with comprehensive pathogen characterization using molecular resistance profiling and CRISPR-based diagnostics to enable rapid strain identification and therapeutic matching. Based on these inputs, tailored bacteriophage strategies are designed, incorporating strictly lytic phages, engineered phages, jumbo phages, and biofilm-disrupting elements. Optimized treatment strategies combine phage therapy alone or in sequence with antibiotics, with careful consideration of administration route, dosing, and timing. At the infection site, phages mediate targeted bacterial lysis, biofilm disruption, attenuation of resistance mechanisms, and suppression of virulence. NIR bioimaging and molecular monitoring provide real-time feedback on phage distribution, replication, and therapeutic response, enabling adaptive adjustment of phage composition and dosing. The lower panel highlights essential translational components, including GMP compliant production, genomic safety screening, and adaptive regulatory pathways. This model depicts an integrated diagnostic therapy monitoring loop that supports rational, data-driven deployment of bacteriophage therapy in clinical settings.

Moving bacteriophage therapeutics from experimental use toward broader clinical application depends on meeting stringent manufacturing and quality standards. Current expectations extend well beyond simple enumeration of plaque-forming units and include full genomic characterization to exclude lysogenic elements, toxin genes, and antimicrobial resistance determinants; validated sterility testing; tight control of endotoxin content; and quantification of residual host DNA and protein contaminants (Schooley et al., 2017; Turner et al., 2021; Chaikeeratisak et al., 2017; Hatfull, 2015; Jault et al., 2019; Merabishvili et al., 2009; Gill and Hyman, 2010; Zschach et al., 2015; Young, 2014). For inhaled formulations, additional attributes such as aerosol performance, particle size distribution, and stability during storage and delivery become central to product consistency (see Figure 1; Table 1).

**Table 1:** Representative bacteriophage therapeutics evaluated against antimicrobial-resistant bacterial pathogens.

Phage/cocktail	Target pathogen(s) and resistance profile	Clinical status	Administration route(s)	Key mechanism and safety notes
BFC1 (ISP + PNM + 14/1)	*Pseudomonas aeruginosa* (MDR/XDR; post-transplant)	Compassionate use	Intravenous; intralesional (early)	Strictly lytic cocktail; endotoxin-controlled; no adverse events; infection resolved
EFgrKN + EFgrNG	*Enterococcus faecium* (vancomycin resistant)	Compassionate use	Intravenous	Lytic phages; GMP-produced; inflammatory markers declined
LBP-EC01	*Escherichia coli* (ESBL, MDR UTI)	Phase II trial	Intra-urethral → intravenous	Engineered lytic phages delivering CRISPR–Cas3; no serious adverse events
PASA16	*P. aeruginosa* (MDR/XDR)	Compassionate use series	Intravenous, topical	Lytic phage therapy; favorable outcomes in ~87%
BA3	*Acinetobacter baumannii* (XDR; VAP)	Compassionate use	Nebulized	Lytic phage; well tolerated; inflammatory markers declined
ISP + BUCT700 + PNM/PT07	MRSA, *Stenotrophomonas*, *P. aeruginosa* (MDR)	Compassionate use	Multiroute (IV, topical, inhaled)	Pathogen-matched lytic phages; full recovery
INF/pB	*P. aeruginosa* (pan-drug resistant; cystic fibrosis)	Compassionate use	Nebulized	LPS-targeting lytic phages; improved lung function
Metamorpho + Mineola + pKp20	*Klebsiella pneumoniae* (ESBL UTI)	Compassionate use	Intravenous	Capsule-targeting lytic phages; durable remission
PP1131 (PhagoBurn)	*P. aeruginosa* (burn infections)	Phase I/II trial	Topical	Safe but limited efficacy due to low titer
DUOFAG^®^	*Staphylococcus aureus*, *P. aeruginosa* (chronic wounds)	Hospital exemption (EU)	Topical	GMP-produced lytic phages; good safety
AP-PA02	*P. aeruginosa* (MDR; cystic fibrosis)	Phase Ib/II	Inhaled	Lytic cocktail; safe pulmonary delivery
AB-SA01	*Staphylococcus aureus* (including MRSA)	Phase I/II	Intravenous, intra-osseous	Lytic myoviruses; well tolerated
TP-102/TP-122A	*P. aeruginosa*; *E. coli*	Phase I/II	Topical; intravenous/intravesical	Broad host-range lytic cocktails
STP-10/NIAID sets	MDR *Staphylococcus*, *Enterobacter* spp.	Expanded access	IV, topical, inhaled	Lytic phages; limited but favorable safety data

There is increasing recognition that traditional potency metrics do not always correlate with clinical performance, particularly in biofilm-associated infections. As a result, alternative benchmarks, including biofilm reduction assays and quantitative measures of phage–antibiotic interaction, are being explored as more informative indicators of therapeutic activity. Regulatory approaches remain variable across jurisdictions. In the United States, bacteriophages are generally evaluated within investigational new drug and biologics frameworks, whereas European pathways are still evolving. The Belgian magistral model has emerged as a practical example of how personalized phage preparations can be supplied under pharmacy oversight using pre-characterized phage collections and standardized quality controls (Jault et al., 2019; Brives and Pourraz, 2020; EMA, 2024; FDA, 2023).

Early clinical initiatives, such as the PhagoBurn program, highlighted practical challenges related to formulation stability and supply logistics. More recent and ongoing studies in settings such as cystic fibrosis and ventilator-associated pneumonia are increasingly incorporating clinically meaningful outcomes, including ventilator-free days, reduction in antibiotic exposure, and relapse rates at defined follow-up intervals, rather than relying solely on short-term microbiological endpoints (Jault et al., 2019; Ferry et al., 2022; Armata Pharmaceuticals, 2022a; Pherecydes Pharma, 2021a).

#### 5.1.8 Clinical development programs and the evolving commercial landscape

A number of biotechnology groups are now translating bacteriophage concepts into structured clinical development programs. Armata Pharmaceuticals is evaluating phage cocktails targeting *Staphylococcus aureus* and *Pseudomonas aeruginosa* across intravenous and inhaled indications, including cystic fibrosis, ventilator-associated pneumonia, and bloodstream infection (Armata Pharmaceuticals, 2022b; Pherecydes Pharma, 2021b; Schooley, 2023). Adaptive Phage Therapeutics has adopted a curated phage-bank approach to support individualized treatment under expanded-access and trial frameworks, with active programs in orthopedic and urinary tract infections (Aslam et al., 2020; Doub et al., 2020; Petrovic Fabijan et al., 2020; Adaptive Phage Therapeutics, 2020; Adaptive Phage Therapeutics, n.d). In Europe, Pherecydes Pharma is pursuing both magistral and clinical pathways focused on *Staphylococcus aureus*, *Pseudomonas aeruginosa*, and *Escherichia coli* (Pherecydes Pharma, 2021c).

Locus Biosciences has advanced bacteriophages enhanced with clustered regularly interspaced short palindromic repeats–associated nuclease activity into early human studies for *Escherichia coli* urinary tract infection, representing one of the first attempts to combine phage therapy with targeted genetic disruption in a clinical setting (Bikard et al., 2014; Citorik et al., 2014; Pursey et al., 2018; Rodrigues et al., 2019; Leitner et al., 2021). Other companies, including Technophage, Intralytix, and Eligo Bioscience, are pursuing indications such as diabetic foot infection, osteomyelitis, and foodborne disease while investing in scalable production and quality systems (Jault et al., 2019; Intralytix Inc., 2019; Hagens and Loessner, 2007; Eligo Bioscience, n.d.; Technophage, 2021). Although fully licensed systemic phage products are not yet available in the United States or European Union, continued clinical use in countries such as Georgia and Poland provides real-world experience that is shaping regulatory discussions and informing future development strategies.

#### 5.1.9 High-value research gaps and strategic priorities

Despite clear momentum, several practical and scientific gaps still limit how confidently bacteriophage therapy can be standardized and compared across studies. First, the field continues to rely heavily on plaque-based measures, plaque-forming units, efficiency of plating, and related readouts, that describe activity under simplified laboratory conditions but often correlate poorly with performance in biofilm-dominant or device-associated infections. What is needed now is broader agreement on assays that better reflect clinical reality, including standardized biofilm reduction or eradication frameworks, structured phage–antibiotic interaction maps, and quantitative *in vivo* response metrics (for example, burden-decay parameters) derived from near-infrared tracking where that approach is used (Latka et al., 2017; Jault et al., 2019; Merabishvili et al., 2009; Gill and Hyman, 2010; Zschach et al., 2015; Young, 2014). These measures would make it easier to compare products, explain heterogeneous outcomes, and select candidates for translation with more confidence.

Second, pharmacokinetic and pharmacodynamic thinking must be adapted to the fact that bacteriophages are self-amplifying and context-dependent. Models that work well for small-molecule antimicrobials often fail to capture the variables that determine phage performance, including bacterial density at the infection site, spatial barriers to diffusion, immune-mediated clearance, emergence of neutralizing antibodies during prolonged exposure, and the extent of replication that occurs *in situ*. Route-specific frameworks are especially important, because intravenous, inhaled, and intravesical delivery each impose distinct constraints on distribution, persistence, and effective exposure at the target site (Comeau et al., 2007; Torres-Barceló et al., 2014; Chaudhry et al., 2017; Oechslin, 2018; Aslam et al., 2020; Doub et al., 2020; Petrovic Fabijan et al., 2020; Shcherbakova et al., 2018). Building these models around clinically relevant sampling strategies, and being explicit about uncertainty, will improve trial design and reduce the tendency to overinterpret early signals.

Third, the evolutionary dimension needs to be approached more systematically. Although “resistance steering” is frequently discussed, the evidence base remains fragmented across pathogens, phage types, and infection contexts. Well-designed resistance mapping under sustained phage pressure, ideally coupled to fitness and virulence phenotyping, would allow the creation of species-specific atlases describing which receptor changes are most likely, how often efflux systems are altered, and what trade-offs are reproducibly observed. Such atlases would directly inform rational cocktail design by shifting decisions from intuition to probabilistic expectation (Hyman, 2019; Chan et al., 2018; Burmeister et al., 2020; Labrie et al., 2010; Westra et al., 2014; Hauser, 2009; Fortaleza et al., 2025).

Fourth, manufacturing remains a central bottleneck, especially when the clinical need is speed, flexibility, and consistent quality at scale. Approaches under active development include modular regulatory documentation, lyophilized or otherwise stabilized phage repositories, improved host-range prediction supported by computational screening, and standardized “swap-in/swap-out” strategies for receptor-binding proteins to expand coverage without rebuilding an entire product from the ground up (Mirzaei and Nilsson, 2015; Jault et al., 2019; Pirnay et al., 2018; Hesse et al., 2020; Brives and Pourraz, 2020; EMA, 2024; FDA, 2023; Russell and Hatfull, 2017). Progress here matters because the most elegant biological concept will still fail clinically if it cannot be delivered reproducibly and safely.

Regulatory alignment will likely depend on pragmatic recognition that curated, quality-controlled phage libraries can be governed under fixed manufacturing and testing standards even when the exact composition is updated to match circulating strains. In parallel, companion diagnostics, particularly those based on clustered regularly interspaced short palindromic repeats systems and near-infrared–enabled monitoring, may become essential for linking a given therapeutic phage choice to a defined bacterial genotype or resistance marker profile, and for documenting response in a way that supports decision-making rather than *post hoc* interpretation (Brives and Pourraz, 2020; EMA, 2024; FDA, 2023). Finally, the imaging layer itself will benefit from continued engineering toward brighter, smaller, and more stable monomeric reporters and stronger photoacoustic performance, particularly for deep infections or low-burden states where signal-to-noise becomes the limiting factor (Filonov et al., 2011; Shcherbakova and Verkhusha, 2013; Piatkevich et al., 2013; Shcherbakova et al., 2015; Ntziachristos, 2010; Shcherbakova et al., 2016; Piatkevich et al., 2017; Shcherbakova et al., 2018; Filonov et al., 2011; Piatkevich et al., 2013; Greenwald et al., 2018; Miao and Pu, 2016; Knox et al., 2017; Kasatkina et al., 2022).

### 5.2 Precision bacteriophage therapy blueprints for key clinical syndromes

#### 5.2.1 Ventilator-associated pneumonia caused by *Pseudomonas aeruginosa* or *Acinetobacter baumannii*

In ventilator-associated pneumonia caused by *Pseudomonas aeruginosa* or *Acinetobacter baumannii*, timing and delivery are often as important as phage selection. A practical starting point is early airway-directed therapy using nebulized bacteriophage cocktails, chosen to cover the patient isolate with multiple, non-overlapping receptor targets. In most settings, three to five strictly lytic bacteriophages provide a reasonable balance between breadth and manufacturing feasibility. For *Pseudomonas aeruginosa*, including a bacteriophage that exploits predictable fitness costs can be advantageous; for example, bacteriophage OMKO1 has been described to select for receptor changes that impair efflux-associated pathways and can shift susceptibility profiles in a direction that helps companion antibiotics. Where available and appropriate, large-genome bacteriophages that perform well in dense biofilm environments (including bacteriophages often discussed in the context of multidrug-resistant *Pseudomonas* infections) may strengthen coverage, but selection should remain isolate-driven rather than name-driven. For *Acinetobacter baumannii*, cocktails that include bacteriophages with polysaccharide-depolymerizing capacity are particularly useful when the clinical isolate is heavily encapsulated or the infection is biofilm-rich, because biofilm weakening improves access for both bacteriophages and antibiotics. If the clinical picture suggests extension beyond the airway, persistent bacteremia, or failure of airway-directed therapy, escalation to intravenous bacteriophage delivery becomes reasonable in principle, with attention to formulation purity and endotoxin limits. Response monitoring should be anchored in clinical trajectory and objective sampling—serial airway sampling when feasible, inflammatory markers such as C-reactive protein, and targeted molecular assays when culture is slow or confounded by prior antibiotics. Imaging-based pharmacology (including near-infrared reporter strategies in research settings) is valuable for defining dosing intervals in models, but in routine care, the closest analogue is consistent, protocolized clinical and microbiological reassessment rather than a single “clearance” time point (Köhler et al., 2023; Chaudhry et al., 2017; Levin and Bull, 1996; Aslam et al., 2020; Doub et al., 2020; Petrovic Fabijan et al., 2020; Van Nieuwenhuyse et al., 2022; Ferry et al., 2022).

#### 5.2.2 Catheter- and device-associated infections

Catheter- and device-associated infections remain a biofilm problem first and a bloodstream problem second, so the strategy should reflect that biology. Source control is central: when device salvage is realistic, a biofilm-directed approach using catheter-lock bacteriophage therapy can deliver concentrations that are difficult to achieve systemically, while limiting whole-body exposure. The most defensible lock formulations use bacteriophages selected for the patient isolate and assembled to avoid single-receptor dependence. Where the organism produces a thick extracellular matrix, choosing bacteriophages with demonstrable extracellular polymeric substance, degrading activity can improve penetration and shorten the time to meaningful biofilm disruption. In practice, lock therapy is best treated as part of a bundle: concurrent systemic antibiotics guided by susceptibility testing, careful management of anticoagulant or lock constituents to avoid inactivating the bacteriophages, and a low threshold for device exchange when clinical improvement is not prompt (Jault et al., 2019; Doub et al., 2020; Morris et al., 2019; Pherecydes Pharma, 2021d).

#### 5.2.3 Complicated urinary tract infections

Complicated urinary tract infections caused by uropathogenic *Escherichia coli* or extended-spectrum beta-lactamase–producing Enterobacterales are well suited to intravesical bacteriophage instillation because the infection space is accessible and high local exposure is achievable. The most important step is rapid isolate matching, ideally through same-day susceptibility screening platforms where available, because urinary isolates are heterogeneous and empiric cocktails can miss key receptor variants. Intravesical delivery can be paired with systemic or oral antibiotics chosen on the basis of susceptibility and tissue penetration, and the sequence can be adjusted depending on symptom burden and bacterial load. Re-dosing is often guided by symptom recurrence and objective urine testing rather than fixed schedules. Gene-targeting bacteriophage strategies that deliver clustered regularly interspaced short palindromic repeats payloads are conceptually attractive for resistance reversal, but at present they should be framed as emerging or investigational rather than routine, and positioned as adjunctive options where regulatory and manufacturing pathways support their use (Bikard et al., 2014; Citorik et al., 2014; Pursey et al., 2018; Rodrigues et al., 2019; Leitner et al., 2021; Kortright et al., 2019; Howard-Jones et al., 2022).

#### 5.2.4 Osteomyelitis and prosthetic joint infections caused by methicillin-resistant *Staphylococcus aureus*

Osteomyelitis and prosthetic joint infection caused by methicillin-resistant *Staphylococcus aureus* demand a combined surgical and antimicrobial plan; bacteriophage therapy, when used, should sit within that framework rather than replace it. Debridement and hardware management remain the foundation, followed by systemic antibiotics with established activity in biofilm-associated staphylococcal disease. Local bacteriophage delivery, through intra-articular irrigation, intraoperative application, or depot-style carriers where available, can provide sustained high concentrations at the site of infection and is biologically plausible for biofilm disruption, particularly when the bacteriophages are strictly lytic and selected against the patient isolate. Courses are typically measured in weeks, with serial clinical evaluation and laboratory monitoring. Because prolonged exposure can provoke neutralizing antibody responses in some patients, it is reasonable to monitor for loss of apparent activity and to consider bacteriophage rotation or cocktail adjustment when the clinical response plateaus without another explanation. Purified bacteriolytic enzymes derived from bacteriophages, including endolysin-based candidates, remain useful to discuss as adjuncts because they can disrupt staphylococcal biofilm structure rapidly and may improve antibiotic penetration; however, clinical positioning should be cautious and evidence-led, acknowledging that trial endpoints and infection phenotypes strongly influence apparent efficacy (Fowler et al., 2024; Schuch et al., 2014; McCarthy, 2024; Fenton et al., 2010; Chan et al., 2013; Tkhilaishvili et al., 2019).

## 6 Conclusion

Bacteriophages are increasingly viewed not as simple substitutes for antibiotics, but as biologically active therapeutics that can be tailored to a pathogen, amplify at the site of infection, and act within biofilms that often defeat conventional regimens. Their clinical value is most plausible when therapy is built on clear design principles: selecting strictly lytic bacteriophages against the patient isolate, combining them in receptor-diverse mixtures to reduce single-step escape, and prioritizing candidates with measurable biofilm-disrupting activity. In parallel, bacteriophage-derived enzymes such as endolysins, as well as gene-targeting constructs that deliver clustered regularly interspaced short palindromic repeats payloads, broaden the toolbox from “killing” alone to more deliberate disruption of resistance determinants and biofilm structure. Importantly, the strongest human evidence to date comes from compassionate-use experiences, case series, and early clinical programs in which bacteriophage therapy, often alongside antibiotics and source control, has been associated with meaningful improvement in infections caused by multidrug-resistant and extensively drug-resistant organisms. These signals are encouraging, but they also underline why careful trial design, isolate-level matching, and clinically relevant endpoints are essential before routine adoption can be justified. The next phase of progress will depend less on enthusiasm and more on infrastructure. Manufacturing pipelines that meet good manufacturing practice expectations, robust genomic screening and release testing, and practical models for personalized preparation, such as magistral compounding frameworks used in Belgium, provide a workable foundation for safe and repeatable deployment. At the same time, the field needs shared performance standards that reflect real clinical barriers. Plaque counts and efficiency of plating alone are not sufficient surrogates for efficacy in chronic, biofilm-dominated infections. Biofilm eradication assays, quantitative maps of bacteriophage–antibiotic interactions, and route-specific pharmacokinetic and pharmacodynamic models for intravenous, inhaled, and intravesical delivery should become routine components of preclinical packages and clinical trial protocols. Diagnostic integration can further strengthen precision: rapid, culture-independent clustered regularly interspaced short palindromic repeats diagnostics, paired with imaging approaches that enable deep-tissue tracking in experimental systems, point toward feedback-guided strategies in which strain identification, therapeutic selection, and treatment monitoring are explicitly linked. If these elements mature together, quality-controlled bacteriophage libraries, standardized analytics, and clearer cross-jurisdictional regulatory alignment, bacteriophage therapy can move from sporadic rescue use to a reproducible, regulated strategy that complements antibiotics and source control in modern infectious disease care. The broader implication is not simply an “extra option” for resistant infections, but a more adaptive therapeutic architecture, designed to function in the biological and evolutionary reality that defines the post-antibiotic era.
